# Wood Decay Characteristics and Interspecific Interactions Control Bacterial Community Succession in *Populus grandidentata* (Bigtooth Aspen)

**DOI:** 10.3389/fmicb.2019.00979

**Published:** 2019-05-09

**Authors:** Eiko E. Kuramae, Marcio F. A. Leite, Afnan K. A. Suleiman, Christopher M. Gough, Buck T. Castillo, Lewis Faller, Rima B. Franklin, John Syring

**Affiliations:** ^1^Department of Microbial Ecology, Netherlands Institute of Ecology, Wageningen, Netherlands; ^2^Department of Biology, Virginia Commonwealth University, Richmond, VA, United States; ^3^Department of Ecology and Evolutionary Biology, University of Michigan, Ann Arbor, MI, United States; ^4^Department of Biology, Linfield College, McMinnville, OR, United States

**Keywords:** microbial community ecology, 16S rRNA, wood decomposition, wood microbiome, interspecific interaction, facilitation

## Abstract

Few studies have investigated bacterial community succession and the role of bacterial decomposition over a continuum of wood decay. Here, we identified how (i) the diversity and abundance of bacteria changed along a chronosequence of decay in *Populus grandidentata* (bigtooth aspen); (ii) bacterial community succession was dependent on the physical and chemical characteristics of the wood; (iii) interspecific bacterial interactions may mediate community structure. Four hundred and fifty-nine taxa were identified through Illumina sequencing of *16S rRNA* amplicons from samples taken along a continuum of decay, representing standing dead trees, downed wood, and soil. Community diversity increased as decomposition progressed, peaking in the most decomposed trees. While a small proportion of taxa displayed a significant pattern in regards to decay status of the host log, many bacterial taxa followed a stochastic distribution. Changes in the water availability and chemical composition of standing dead and downed trees and soil were strongly coupled with shifts in bacterial communities. Nitrogen was a major driver of succession and nitrogen-fixing taxa of the order Rhizobiales were abundant early in decomposition. Recently downed logs shared 65% of their bacterial abundance with the microbiomes of standing dead trees while only sharing 16% with soil. As decay proceeds, bacterial communities appear to respond less to shifting resource availability and more to interspecific bacterial interactions – we report an increase in both the proportion (+9.3%) and the intensity (+62.3%) of interspecific interactions in later stages of decomposition, suggesting the emergence of a more complex community structure as wood decay progresses.

## Introduction

Decomposition of coarse woody debris (CWD) – large diameter wood that has senesced – is an essential process linked to the cycling of carbon and nutrients in forest ecosystems (Prewitt et al., 2014; Kielak et al., 2016b). The process of decomposition is largely governed by the combined action of bacterial and fungal communities – where bacteria play an important role in breaking down complex organic molecules such as cellulose, hemicellulose, lignin, and soluble sugars (McGuire and Treseder, 2010), as well as increasing wood permeability (Clausen, 1996). However, despite the well-recognized importance of bacteria, little is known about how their community structure changes as decomposition progresses and substrate quality changes.

Many competing theories and hypotheses exist to explain how organisms assemble during the process of community succession, with most theoretical frameworks and observations focusing on the role of resource and habitat changes in plant systems (Zhang, 2014). To the extent that microbial ecologists have considered these issues, the focus has been theoretical with limited empirical testing (Dini-Andreote et al., 2015). Though explicit studies of bacterial community succession are rare, prior research documenting a strong relationship between bacterial community structure and wood chemical and physical properties (Hoppe et al., 2014; Kielak et al., 2016b) suggest substantial changes in community structure occur as wood decomposition progresses. Prior studies have shown that decreases in wood density and remaining mass, increases in relative moisture, decreases in the carbon:nitrogen (C/N) ratio, and changes in the concentration of N and phosphorus (P) are likely to be especially important drivers (Johnson et al., 2014). These sorts of directional changes in wood characteristics as decomposition progresses provide a suitable study system to evaluate the variable selection scenario proposed by Dini-Andreote et al. (2015) where the emergence of new ecological niches follows from the changes in environmental conditions. Moreover, succession along a gradient of wood decomposition provides an interesting environment to examine interspecific bacterial interactions. In this respect, changes in the bacterial community may reflect on community interactions (complementary niches, competition, and facilitation), but we still lack studies able to disentangle these interspecific interactions from abiotic interactions – where two taxa do not interact with each other, but rather both are dependent on the same environmental factor.

To investigate the mechanisms that govern bacterial community assembly, a chronosequence of wood decay was identified, in which each log represents a discrete habitat unique from the soil environment, and where decay classes of each log can be used to demarcate stages along the continuum of succession. In this study, we examined bacterial community structure and succession in standing and downed *Populus grandidentata* Michx. (bigtooth aspen). Our model system centers on *P. grandidentata* for two reasons. First, *Populus* is among the most abundant and broadly distributed woody plant genera globally (Hamzeh and Dayanandan, 2004). Secondly, *P. grandidentata*’s decay status in our study site, spanning from standing dead to downed and extensively decomposed CWD, offered a complete successional continuum for examining bacterial community structural changes. Our primary objectives were to: (i) determine how bacterial diversity and abundance changed along a gradient of decomposition; (ii) identify how changes in the chemical and physical properties of CWD drive bacterial community succession; and (iii) investigate how interspecific bacterial interactions may mediate community structure.

## Materials and Methods

### Study Site

The study was conducted at the University of Michigan Biological Station in northern lower Michigan, United States (45°35.5′N, 84°43.0′W). The average annual (1942–2014) temperature for this site is 5.8°C and the average annual precipitation is 782 mm. CWD samples were identified within a long-term sampling plot covering 1.13 ha (60 m fixed radius). The plot lies on a high-level glacial outwash plain with soils that are primarily excessively drained sands of the Rubicon-East Lake series.

The dominant forest type is a 100-year-old secondary successional mixed northern hardwood forest that naturally regenerated following clear-cut harvesting and fire in 1911 (Gough et al., 2007a). This forest is rapidly transitioning from the early to middle stages of ecological succession. The forest canopy averages 22 m in height, and upper canopy tree species primarily include rapidly declining early successional *P. grandidentata* and *Betula papyrifera* Marsh. (paper birch), which are being supplanted by later successional *Quercus rubra* L. (northern red oak), *Acer rubrum* L. (red maple) and *Pinus strobus* L. (white pine) (Schmid et al., 2016). The understory consists of *Pinus strobus* L., as well as a minor component of *Fagus grandifolia* Ehrh. (American beech). Stem density of trees ≥8 cm diameter at breast height (dbh) is 700–800 ha^-1^, basal area is ∼25 m^2^ ha^-1^, and leaf area index (LAI) averages 3.5. The groundcover is dominated by *Pteridium aquilinum* L. (bracken fern) with seedlings of *A. rubrum*, along with *Gaultheria procumbens* L. (wintergreen), *Acer penslvanicum* L. (moosewood), *Maianthemum canadense* Desf. (wild lily of the Valley), *Vaccinium angustifolium* Ait. (blueberry), and *Trientalis borealis* Raf. (starflower).

### Field Sampling

Within the confines of the 60-m fixed radius-plot, we collected samples from 24 *P. grandidentata* individuals at various stages of decay as well as three soil cores in July, 2015. Four of the *P. grandidentata* were standing dead (SD), and the remaining 20 individuals had fallen to the forest floor as a result of natural causes and were comprised of four replicates from each of five decay classes. Decay classes (DC) were assigned based on visual and mechanical inspections, with DC1 showing minimal signs of decay and DC5 showing highly advanced levels of decay (Marra and Edmonds, 1994). *Populus grandidentata*, which maintains its distinct bark texture through very late decay stages, was identified following protocols previously used at our site (Gough et al., 2007b; Schmid et al., 2016). Sampled SD trees and CWD were distributed roughly evenly across the plot. All samples were identifiable as *P. grandidentata* and met the criteria of: (i) >3 m in length, (ii) >15 cm in diameter, (iii) in direct contact with the forest floor (with the exception of SD), and (iv) >10 m from either side of the access trail. Using these criteria, we established a chronosequence that encompasses all stages of decay starting with standing dead trees, following through each of the five stages of decomposition (DC1 through DC5), and ending in soil.

We obtained wood samples using a 19.05 mm ethanol-sterilized drill bit. Before drilling, extraneous leaf litter and dirt were removed from the log; however, bark remaining on the log was not removed in an effort to best represent the whole-log decomposer community. For each piece of CWD included in our analysis, wood samples were taken from three radial locations positioned at three separate intervals for a total of nine samples per log in an effort to account for variation across a log (Supplementary Figure S1). Drilling points were taken at least 0.5 m from the end of the log and 0.5 m away from each other. The diameter of the logs at each sampling point was recorded and holes were drilled to a depth of approximately 7 cm. Samples from standing dead trees were taken at 0.9, 1.4, and 1.9 m, and at azimuths of 120, 240, and 360 degrees around the tree from the ground. Once samples were drilled, wood was removed with ethanol-sterilized forceps and placed into large plastic bags. Soil samples were taken 30 m out from plot center at azimuths of 120°, 240°, 360°. The top 5 cm of soil (predominantly O and A horizons) were collected into a large plastic bag at each point.

### Sample Processing, DNA Extraction, and Sequencing

For chemical analysis, the wet weight of each drill sample was recorded in the lab. Each sample was then lyophilized and weighed again to determine moisture content, which is reported as the average of nine samples for each log or tree. A composite sample was then made for each log or tree by combining a 1 g subsample of lyophilized material from each of the nine drill points. To ensure sample homogeneity, the composite samples were ball-ground for 5 min with a SPEX 800D mixer/mill (SPEX Sample Prep., Metuchen, NJ, United States) to a sawdust consistency. To avoid contamination, the mixer/mill was sterilized with 70% ethanol between samples in addition to thorough cleansing of the chamber. These composite samples were stored at -80°C until chemical analyses were performed.

For genomic work, a composite sample was made for each log or tree by combining a 1 g subsample of ground, lyophilized sample from each of the nine drill points. The composite samples were ball-ground to a sawdust consistency as above. Prior to and following grinding, samples were stored at -80°C. Genomic DNA was extracted from 80 mg of each composite sample using the PowerMax Soil DNA Isolation Kit (MoBio, Carlsbad, CA, United States). To increase homogenization, 0.2 g of garnett was included in each extraction tube. Standard manufacturer protocols were followed except that samples were vortexed for 25 min instead of using a PowerLyzer for the homogenization step. DNA was quantified on a Qubit fluorometer using high sensitivity (HR) chemistry (Thermo Fisher Scientific, Waltham, MA, United States).

Extracted DNA was used as a template to amplify the *16S rRNA* gene V5–V7 variable region using PCR primers 799F (barcoded) and 1193R (Supplementary Table S1). Amplification proceeded in a 30 cycle PCR using the HotStarTaq Plus Master Mix Kit (Qiagen, Hilden, Germany) with initial denaturation at 94°C for 3 min, followed by 28 cycles of denaturation at 94°C for 30 s, annealing at 53°C for 40 s and extension at 72°C for 1 min, and a final elongation at 72°C for 5 min. The quality of PCR products was assessed on 2% agarose gels to determine the success of amplification and the relative intensity of bands. Multiple barcoded samples were pooled in equimolar ratios based on their DNA concentrations. Pooled samples were purified using calibrated Ampure XP beads (Beckman Coulter, Brea, CA, United States) for use in library preparation. Pooled and purified PCR product was used to prepare a DNA library by following the Illumina TruSeq DNA library preparation protocol (Illumina, San Diego, CA, United States). Sequencing was performed at MR DNA (Shallowater, TX, United States) on a MiSeq 2 × 300 bp Version 3 following the manufacturer’s guidelines.

### Sequence Analysis, Taxonomic Classification, and Data Submission

A standardized pipeline including quality filtering, clustering, and taxonomic annotation was applied for the bioinformatic analyses. Sequence data quality control and processing used a combination of the UPARSE and QIIME pipelines (Caporaso et al., 2010; Edgar, 2013). Briefly, after removing the adaptors and primer sequences, sequences were demultiplexed by unique barcodes. Then, using USEARCH 7, sequences with a maximum expected error of 0.5 or a length shorter than 250 bp were removed, dereplicated, clustered to operational taxonomic units (OTUs) with a threshold of 97% identity, and filtered for additional chimeras. Using the UCLUST method, taxonomy was assigned to representative *16S rRNA* sequences using the Greengenes database (DeSantis et al., 2006) at 97% similarity. Sequences were aligned using the PyNAST algorithm. The resulting OTU table was converted to Biological Observation Matrix (BIOM) format (McDonald et al., 2012). All samples were rarefied (generated at a depth of 24,804 sequences) for alpha diversity analysis. Sampling effort was estimated by Good’s coverage (Good, 1953). The *16S rRNA* sequence data are available at the European Nucleotide Archive (ENA)^1^ under the study accession number PRJEB26754.

### Chemical Analysis

We conducted chemical analyses to evaluate changes in wood quality and nutrient cycling with advancing decomposition. Wood percent C and N, and δ15N of milled composite samples were derived using a Costech ECS 4010 elemental analyzer interfaced with a Thermo Scientific Delta Plus XP isotope ratio mass spectrometer in the analytical chemistry lab at the University of Michigan Biological Station (Pellston, MI, United States). Overall external precision for %C was 0.20% of measured values, %N was 0.15% of measured values, and δ15N was 0.02 per mill. pH was collected on 0.2 g of composite, ball-ground material from each log or soil sample vortexed for 5 min in 2 ml of distilled water (Model 8000 pH meter, VWR Scientific, Radnor, PA, United States).

### Statistical Community Analysis

We assessed community structure using rarefaction-based alpha-diversity metrics via phylogenetic diversity, Chao1 estimator, and Shannon diversity calculated using QIIME 1.9 (Caporaso et al., 2010).

The changes in bacterial community composition along the chronosequence were determined by multivariate regression tree (MRT) analysis (De’ath, 2002) in the “mvpart” R package, and the distance matrix was based on Bray–Curtis built by the function “gdist.” MRT analysis determines linear and non-linear relationships between community composition and provides a set of explanatory variables without requiring residual normality. Prior the MRT analysis, the OTU table was log transformed and the Bray–Curtis distance matrix was calculated to generate the tree after 500 cross-validations and the PCA of the MRT was plotted using the function “rpart.pca” in the “mvpart” package.

The bacterial community within our dataset was characterized as having an overdispersal variance as revealed through evaluation of the mean–variance relationship. Therefore, we applied a generalized linear model (GLM) based on negative binomial distribution which provided the best model fit to investigate the effects of wood decay on taxa abundance. In order to avoid sequencing bias common in next-generation platforms (Lee et al., 2012; McMurdie and Holmes, 2014), we decided to use the total number of reads per sample as a covariance effect in our GLMs. The effect of the treatments in the microbial community was evaluated by the Wald’s test (W) followed by multiple comparisons with the Tukey–Kramer test.

Next, we applied a generalized regression model in a negative binomial distribution for obtaining ecological niche-modeling. This approach allowed us to generalize the multiple regression model that uses the so-called “link” function to accommodate response variables (Austin et al., 1990). We applied the LASSO (least absolute shrinkage and selection operator) penalty as the criteria for both variable selection and regularization to provide a more accurate and interpretable model (Osborne et al., 2000). This procedure provided the standardized regression coefficients that determined the influence of the environmental variable on each bacterial taxon. All analyses were performed at the level of Family in the Linnaean hierarchy.

To investigate habitat sharing, we first filtered all the data by selecting only the taxa found in soil or in standing dead wood. For soil taxa, we obtained a list of bacteria that were identified in the soil and then evaluated how their relative abundance changed over the other identified decay classes. We performed the same analysis with bacterial taxa identified in standing dead trees (SD treatment).

Finally, to investigate the co-occurrence of bacterial taxa, we performed a residue correlation analysis in a latent multivariable model to investigate correlation among groups of bacteria (Order level) without the dependence of wood characteristic variables. To determine the stages of decomposition, we performed model-based simultaneous clustering and ordination as proposed by Hui (2017) using CORAL (Clustering and Ordination Regression Analysis). This method is designed for non-normal responses and uses species-specific rather than cluster-specific factor loadings (regression coefficients). The advantage of this approach is that it simultaneously performs ordination and clustering. Therefore, CORAL classifies samples based on their position as given by the latent variable regression. Using this approach, we assessed our data set for different numbers of clusters and evaluated the model fit using information criteria analysis (AIC, BIC, etc.). This analysis allowed us to determine that we had two clusters in our data set (Supplementary Table S2) corresponding to two stages of decomposition (Supplementary Figure S2): early stage (SD, DC1, DC2) and late stage (DC3, DC4, DC5). To improve visualization, we performed a cluster analysis with these correlation coefficients to determine bacterial taxa that responded similarly to environmental factors. To investigate the difference in the coefficients of correlation, we ran an ANOVA followed by Tukey–Kramer multiple comparison tests. Different levels of significance were based on *p*-values after correcting for a false discovery rate (Benjamini and Hochberg, 1995). The analyses were performed in R using the packages “mvabund” (Wang et al., 2012), “boral” (Hui, 2016), “pvclust” (Suzuki and Shimodaira, 2006), and “multcomp” (Hothorn et al., 2008).

## Results

### Bacterial Diversity and Abundance

Our analysis returned a high sequence density per sample across decay classes and soils. After quality filtering the raw reads, a total of 1,236,465 partial 16S rRNA gene sequences were generated from the 27 samples, with an average of 45,795 sequences per sample (ranging from 24,804 to 80,257). All treatments presented comprehensive sampling of the bacterial community with high sequence coverage (Good’s sequence coverage > 99%; Supplementary Table S3). The quality filtered raw reads were able to be assigned to a total of 2,054 OTUs, and 98.2%, 85.5%, 59.3%, and 25.2% were able to be taxonomically assigned to phyla, order, family, and genus, respectively. Across the three soil samples, 19 different phyla and 53 different classes of bacteria were observed; for SD and DC1–DC5 these values were 23 and 62, respectively.

**FIGURE 1:**
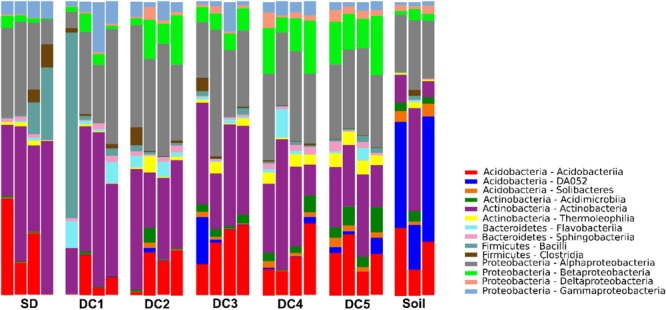
Bar chart showing relative abundance of the dominant bacterial classes in different stages of CWD (SD, DC1, DC2, DC3, DC4, DC5) and soil.

Species richness across all SD and CWD samples ranged from 511 to 1,099 OTUs, with an average of 765.2. Species richness across all soil samples ranged from 620 to 997 OTUs, with an average of 748.0. Alpha diversity indices indicate a progressive increase in bacterial community diversity as decomposition progresses, with peak diversity occurring in the most advanced decay class (DC5; Supplementary Figure S3). This increase was significant for phylogenetic diversity and the Chao1 estimator of richness, but not for Shannon’s index. While diversity appears to drop in the soil relative to DC 5 logs, this decline is not supported statistically in any of the measures of diversity. The relative abundances of dominant taxa varied substantially across the gradient of CWD decomposition (Figure 1). The dominant classes (>1%) across all samples belonged to the phyla Acidobacteria, Actinobacteria, Bacteroidetes, Firmicutes, and Proteobacteria.

### Bacterial Community Succession in Relation to Wood Decay and Chemistry

**FIGURE 2:**
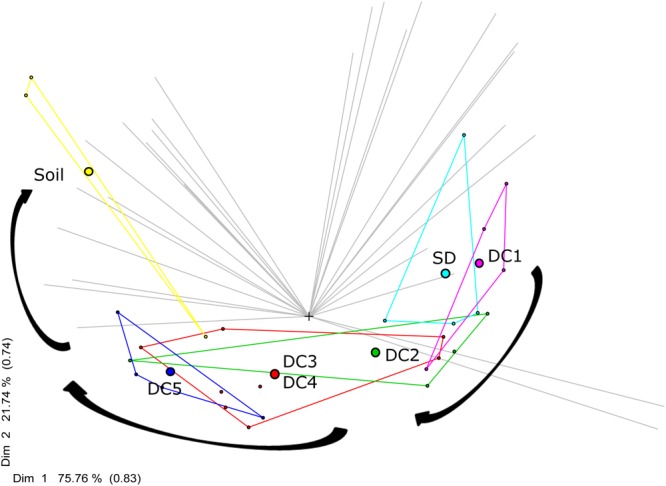
MRT analyses of microbial community succession from standing dead (SD), through wood decay classes (DC1 = Decay Class 1, DC2 = Decay Class 2, DC3 = Decay Class 3, DC4 = Decay Class 4, DC5 = Decay Class 5), and into soil. Filled circles represent the centroid (mean of the multidimensional scale) of microbial community of each decay class. The community of bacteria inhabiting DC3 and DC4 logs were very similar and as a result they were clustered together (red color). The gray lines represent the OTUs. The first two dimensions Dim 1 and Dim 2 account for 75.76% and 21.74% of the between-groups sums of squares, with intersect correlations of 0.83 and 0.74.

The bacterial community changed along the gradient of wood decomposition; in particular the relative abundances of dominant taxa varied between standing dead trees, CWD decay classes, and soil samples. The PCA ordination based on MRT analysis (Figure 2) accounts for 97.5% of the variation in the bacterial community dissimilarity (DIM1 + DIM2, Bray–Curtis distance) and highlights the successional changes in bacterial communities along the decay classes continuum, from standing dead wood to soil.

**FIGURE 3:**
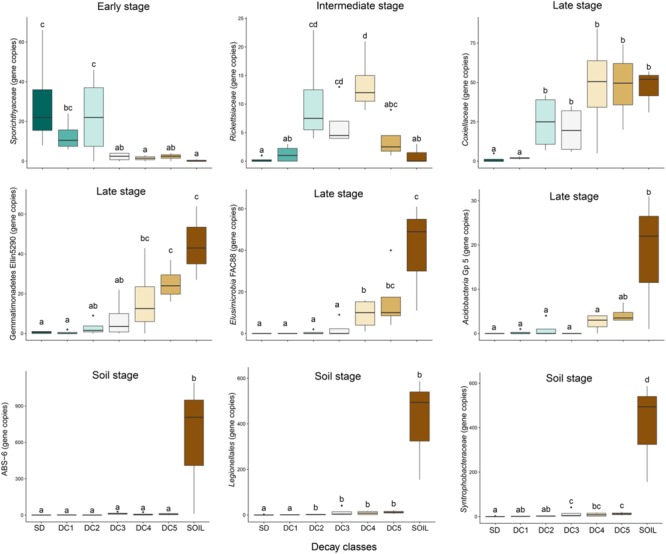
Succession of bacterial groups from standing dead (SD), through wood decay classes (DC1 = Decay Class 1, DC2 = Decay Class 2, DC3 = Decay Class 3, DC4 = Decay Class 4, DC5 = Decay Class 5), and into soil. These nine bacterial taxa were significantly (*p* < 0.05) affected by the decay status of their host logs as suggested by the analysis of variance in a generalized linear model with negative binomial distribution. Boxes represent the 25th to 75th percentiles and display the median values (bold line). The whiskers highlight the maximum and minimum values (non-outlier range). Decay classes followed by the same letter do not differ from each other according to the Tukey–Kramer test with α = 0.05. The *Y*-axis represents bacterial group abundance (estimated gene copy number after correcting for sample sequencing bias).

After correcting for false discovery rates (*q* < 0.05), we found nine bacterial taxa were significantly (*p* < 0.05) affected by the decay status of their host logs (Figure 3). These nine taxa display four generalized patterns over the gradient of decomposition and are named according to where they displayed peak abundance: early stage (decreasing in abundance), intermediate stage (peak at intermediate decay classes), late stage (increasing in abundance), and soil only. *Sporichthyaceae* was more abundant in the initial decay classes associated with an early stage (SD, DC1, and DC2) and then decreased as decomposition progressed; *Rickettsiaceae* peaked in the intermediate decay classes. Four of the nine taxa display a late stage pattern of increasing abundance along the decomposition gradient (*Acidobacteria* Gp5, *Elusimicrobia* FAC88, *Gemmatimonadetes* Ellin5290, *Coxiellaceae*); three of these taxa occurred almost exclusively in the soil (ABS-6, *Legionalles*, and *Syntrophobacteraceae*).

When we included the nearly significant results (*p* < 0.06), 16 additional taxa displayed changes in abundance along the gradient of decomposition. Three of these 16 taxa followed an early stage pattern (Supplementary Figure S4: *Friedmanniela, Methylocystaceae*, and *Rhizobiaceae*), five displayed an intermediate stage pattern (Supplementary Figure S5: *Asticcacaulis biprosthecium, Alicyclobacillaceae, Beijerinckiaceae, Deltaproteobacteria* MIZ46, and *Sporichthya*), and six of these taxa exhibited a late stage pattern (Supplementary Figure S6: *Candidatus solibacter, Gemmatimonadales, Gemmatimonadales* KD8.87, *Mycobacterium celatum, Pedosphaerales*, and *Solibacterales*). The final two of these 16 taxa were largely abundant in the soil only (Supplementary Figure S7: *Koribacteriaceae*, TM1). Many of the taxa showing either an increase or a decrease in abundance corresponding to the decay class (Figure 3 and Supplementary Figures S4–S6) responded to wood decay in two distinct phases: an early stage of bacterial succession corresponding to SD, DC1, and DC2, likely representing a bacterial community more related to the original tree tissue microbiome, and a later stage of bacterial succession corresponding to DC3, DC4, and DC5 that showed a strong convergence toward the soil microbiome.

**FIGURE 4:**
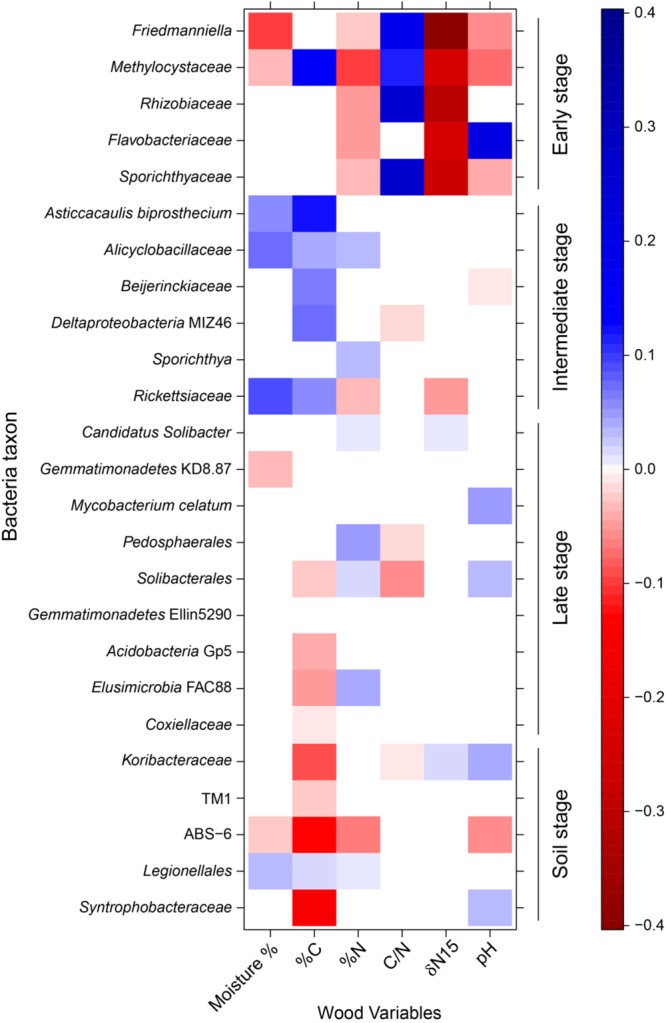
Bacterial taxa abundance modeling results. Standardized coefficients for all environment–taxa interaction terms are presented from a generalized linear models (GLM)-LASSO model. Brighter squares show stronger associations than paler ones, positive associations are blue and negative associations are red. Here, we used all 25 bacterial taxa shown to have statistically significant change (*p* < 0.06; see text) along the decomposition gradient.

Changes in the water availability and chemical composition of standing dead and downed trees and soil along the continuum of decay (Supplementary Figure S8) were strongly coupled with shifts in bacterial communities (Figure 4). In our analysis, 24 of 25 bacterial taxa evaluated (*p* < 0.06; see above) were significantly correlated with the nutrient status, pH, and/or moisture content of decaying wood, properties which changed over the succession of wood decay. Percent C and N correlated with the abundance of 15 and 14 groups, respectively, though the sign of the relationship—positive or negative—varied substantially among taxa. Groups from the *Rhizobiales* order (*Methylocystaceae* and *Rhizobiaceae*) diminished in abundance with increasing %N and δN15, declining as wood decay became more advanced. Changes in pH played a mixed role in the distribution of our selected taxa, with five bacterial taxa increasing and another five decreasing in abundance with rising pH. Many taxa shifted their abundance along multiple chemical and environmental gradients. For example, six of eight taxa that shifted abundance with wood moisture content also exhibited changes with either %N or pH.

With respect to habitat sharing between soil, standing dead trees, and the five classes of decaying wood, we found that a total of 236 taxa were shared between soil and the remaining classes, while 238 taxa were shared with standing dead trees and the remaining classes. Of these totals, however, most taxa were rare, leaving only 15 and 22 taxa shared with soil and standing dead trees, respectively, at abundances greater than 1%. In terms of relative abundance, ∼65% of the bacterial abundance in DC1 logs comes from taxa shared with standing dead trees (Supplementary Figure S9), versus ∼16% shared with soil (Supplementary Figure S10). These shared taxa between standing dead trees and DC1 logs are largely from the phyla Firmicutes, Actinobacteria, Acidobacteria, and Proteobacteria.

### Community Interspecific Interactions

**FIGURE 5:**
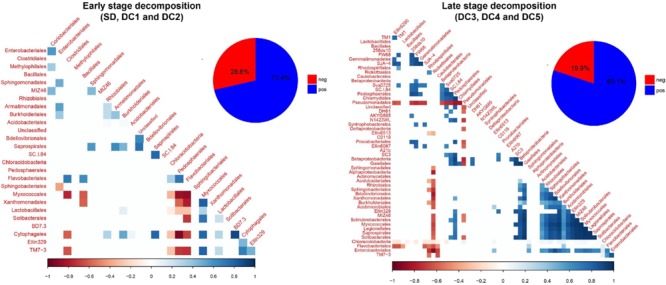
Residual correlation matrix, based on fitting the latent variable model to aspen wood decay. Bacteria are ordered according to their class taxonomic level. Blue squares refer to positive associations while red squares correspond to negative associations. The lighter the square, the weaker the coefficient. Only correlations in which credible intervals do not cross zero are shown.

**FIGURE 6:**
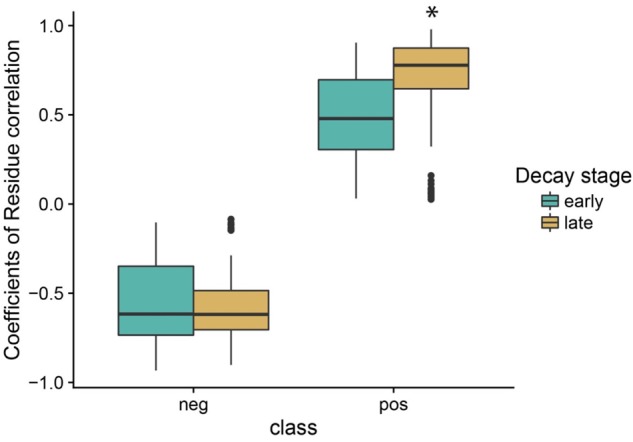
Changes in the coefficient of residue correlation (positive and negative) based on fitting the latent variable model to aspen wood decay. The early stage of decomposition includes standing dead trees and decay classes 1 and 2. The late stage of decomposition includes decay classes 3, 4, and 5 coarse woody debris. The asterisk indicates a statistical difference at 5% probability according to the Tukey–Kramer test for different number of replicates.

We identified two broad stages of bacterial succession, an earlier stage and a later stage, based on (1) patterns of taxa abundance that showed a significant relationship to wood decay class (Figure 3 and Supplementary Figures S4–S7), and (2) the cluster analysis (Supplementary Table S2). In the earlier stage of decomposition (SD, DC1, and DC2), the analysis of residual correlation from the generalized latent multivariate models identified a total of 27 bacterial taxa which showed significant interspecific interactions. Of these interspecific interactions which resulted in a change in abundance, 71.4% showed a significant positive relationship (Figure 5). In the later stage of decomposition (DC3, DC4, and DC5), we found 52 bacterial taxa (Orders) that showed significant interspecific interactions, with a higher percentage of positive interactions (80.1%; Figure 5). In the early stage of decomposition, there is a cluster of negative interactions grouping Sphingobacteriales, Myxococcales, Xanthomonadales, Lactobacillales, Solibacterales, Cytophagales, TM7-3, Enterobacteriales, Clostridiales, Bacillales, Chloracidobacteria, Pedosphaerales, and Flavobacteriales. In the late stage of decomposition, the bacterial group Pseudomonadales represented the majority of significant negative interactions with 23 other bacteria groups; the Rickettsiales also interacted negatively with 18 other groups of bacteria. We also highlight the groups Acidobacteriales, Rhizobiales, Sphingobacteriales, Bdellovibrionales, Xanthomonadales, Burkholderiales, Acidimicrobiales, Ellin329, MIZ46, Solirubrobacterales, Myxococcales, Legionellales, Saprospirales, and Solibacterales as interacting positively with each other. Moreover, when we evaluated the coefficient of correlation we found a significant increase (*p* < 0.05, *q* < 0.05) in the positive interactions between bacterial groups at the late stage of decomposition (Figure 6).

## Discussion

Our understanding of microbial decomposition of CWD remains in its infancy (Baldrian and Banin, 2016). While the role of fungi in this process has received the greatest attention, the role of bacteria, either alone or in conjunction with fungi, has received relatively little attention. The most relevant work on bacterial community succession in CWD has been conducted in Europe on *Picea abies* (Hoppe et al., 2014; Rinta-Kanto et al., 2016), *Pinus sylvestris* (Kielak et al., 2016b), and *Fagus sylvatica* (Hoppe et al., 2014). Given that the details of bacterial community succession are most assuredly ecosystem and tree species specific (Hoppe et al., 2014; Sun et al., 2014), a higher order understanding of the process requires that we investigate additional tree species occurring in unique ecosystems. While studies on bacterial community succession are indeed limited, it is interesting to note that generalizations of the process are beginning to emerge (Hoppe et al., 2014; Kielak et al., 2016b; Rinta-Kanto et al., 2016), as discussed below.

The data presented in this paper establish a pattern of bacterial succession across a chronosequence of aspen decay (Figure 1, 2). As decay progressed, the abundance and diversity of the resident bacterial communities changed in response to shifting resource availability and interspecies interactions (Supplementary Figure S8 and Figure 4). While bacterial succession has been documented in recently deglaciated soils (Sigler et al., 2002), on leaf surfaces (Redford et al., 2010), and even on human corpses (Hyde et al., 2014), few studies have documented bacterial succession over the course of wood decay using a chronosequence approach (Hoppe et al., 2014; Kielak et al., 2016b; Rinta-Kanto et al., 2016). While the particular species of bacteria are largely unique among these studies, members of the phyla Acidobacteria, Actinobacteria, Alphaproteobacteria, and Betaproteobacteria are dominant members across these systems and are found in similar relative abundances (Figure 1), suggesting a common functional role for these groups of bacteria across tree species. Sun et al. (2014), using a buried wood block approach, documented bacterial establishment and community succession occurring over a time frame of only 4 months. In our study system, we estimate the elapsed time from DC1 to DC5 in *P. grandidentata* CWD at 20–30 years, while in other systems with larger trees or with more decay-resistant trees the substrate could be present for a century or more. Our study has shown that the microbiome of standing dead trees (and likely living trees) contributes to patterns of bacterial community establishment and succession, and thus the complete time frame for succession begins with the microbiome of living trees and extends until the CWD is incorporated into soil organic matter.

Measures of bacterial species diversity increased over the course of succession with peak diversity in the later decay stages and soil being significantly different than species diversity in standing dead and DC1 logs (phylogenetic diversity and Chao1; Supplementary Figure S3). Hoppe et al. (2014) and Kielak et al. (2016b) also found increased measures of alpha diversity over a gradient of decomposition, and Rinta-Kanto et al. (2016) documented an overall increase in bacterial abundance over a gradient of decomposition. This generalized pattern of increasing species diversity over the course of succession is consistent with that observed in plant terrestrial ecosystems, in which progressive diversification of habitat and resource distribution over time results in greater niche partitioning and, consequently, plant diversity (Gleason, 1926). Prewitt et al. (2014), who investigated bacterial succession in loblolly pine (*Pinus taeda* L.) and western red cedar (*Thuja plicata* Donn ex D. Don), found that bacteria formed only a minor component of the microbial diversity within decaying wood, which stands in stark contrast to the findings in this paper and related studies (e.g., Zhang et al., 2008; Kielak et al., 2016b), suggesting their methods resulted in an underrepresented bacterial sequencing pool.

We identified a total of 459 taxa present across all standing trees, decaying logs, and soil samples. Of these 459, a total of 317 taxa showed no significant pattern of abundance relative to decay class or soil. We found a total of 142 taxa that displayed a significant pattern across the chronosequence, however, the false discovery rate adjusted that number down to 25 taxa (including all with *q* < 0.06). These 25 taxa largely range from rare to mid-abundance, rather than the most abundant taxa. This suggests that the most abundant taxa are more resilient to changes in environment and community structure. As only 5–6% of the taxa in our study showed a significant pattern across the chronosequence, it seems likely that many bacterial taxa follow a stochastic distribution in regards to decay status. The 25 taxa that were significantly affected by the decay status of their host logs or whose presence was restricted to the soil (Figure 3 and Supplementary Figures S4–S7) displayed peak abundances in four general stages – early, intermediate, late, and soil.

Bacteria displaying an early pattern of abundance were found to be relatively common in standing dead trees and CWD in the first two stages of decay, but then dropped off precipitously as decay progressed. CWD in these stages of decomposition is characterized by a high C/N ratio and a negative δN15 (Supplementary Figure S8 and Figure 4). Five taxa showed an early stage pattern of abundance (Figure 3 and Supplementary Figure S4), which include known nitrogen-fixing bacteria (*Rhizobiaceae, Methylocystaceae*, and *Flavobacteriaceae*) (Sadowsky and Graham, 1998; Nazaries et al., 2013; Bowman, 2015) and other bacterial groups (*Sporichthyaceae, Friedmanniella*) whose presence might represent the native bacteria inhabiting aspen wood or pioneer bacteria tolerant of the high C/N environment. These early colonizing bacterial species are analogous to pioneer species in plant successional pathways that have the ability to tolerate extreme environmental conditions and/or low resource availability and in the process, alter site conditions such that community turnover is favored (Finegan, 1984).

One question of interest related to the bacterial succession within CWD is how bacteria are inoculating the downed wood – some taxa may be part of the original living tree microbiome (Hacquard and Schadt, 2014), some taxa may be delivered via association with fungal mycelial (van der Heijden et al., 2008), and/or some come via direct inoculation from the soil (Clausen, 1996). Our data clearly show that the taxa inhabiting newly downed wood (DC1) are largely derived directly from the microbiome of standing dead trees (and likely living trees) and not the soil (Supplementary Figures S9, S10), and thus the complete time frame for succession begins with the microbiome of living trees. As the path of bacterial succession and associated decomposition are likely determined by those species inhabiting the first stages of downed wood, this suggests a greater emphasis needs to be placed on understanding the composition and establishment of tree microbiomes.

Prior research in other species of *Populus* has shown that the wood microbiome of living trees contains numerous diazotrophic bacteria (Hacquard and Schadt, 2014). The strongly negative values of δN15 in the early stages of decomposition are typical of isotope discrimination by nitrogenase in free-living diazotrophs which undergo preferential reduction of 14N (Unkovich, 2013). Thus, both the presence of N-fixing taxa along with the uncovered δN15 pattern suggest that N-fixation is occurring in our system and may be critical in the early phases of decomposition, providing nitrogen to other bacterial and fungal species that follow in succession. Nitrogen fixation in dead wood has been shown to be significant at the ecosystem level, with estimates ranging up to 2.1 kg fixed N ha^-1^ y^-1^ (Brunner and Kimmins, 2003). N-fixing taxa found in decomposing *Pinus sylvestris* showed a similar early pattern of abundance (Kielak et al., 2016b). As mineralization continues and as fungal species likely translocate nitrogen into the CWD (Baldrian and Banin, 2016), the %N increases resulting in a reduced C/N ratio. Thus, the niche for N-fixing taxa is reduced and these species are succeeded by other taxa. The less negative δN15 values are likely a result of a reduced dependence on N-fixation and an N14:N15 ratio more similar to atmospheric conditions that results from mineralization and translocation. It is also possible that microbially mediated fractionations or preferential retention of 15N enriched substrates is occurring as decomposition progresses, as has been hypothesized to explain isotopic enrichment during litter decay (Connin et al., 2001; Asada et al., 2005). In contrast to our findings, Hoppe et al. (2014) found that N-fixing taxa of the order *Rhizobiales* were more abundant in the intermediate and late stages of decomposition in *Picea abies* and *Fagus sylvatica* suggesting that variation may exist across systems. Another interesting finding associated with the early stage of decomposition is the presence of the family Methylocystaceae, which include known methanotrophs (Bowman, 2015). It has been shown that the anaerobic environments associated with the heartwood of live trees can create conditions that favor methanogenesis, which could then support methanotrophs (Hacquard and Schadt, 2014; Wang et al., 2016). A recent paper by Warner et al. (2017) found that trees are a net methane source, but switch to a sink at some point during the process of decomposition. Our data from *Populus grandidentata* suggest that the switch from source to sink occurs prior to any significant decomposition in the pool of CWD, as members of Methylocystaceae are found nearly exclusively in standing dead trees (Supplementary Figure S4), a result with important implications for the cycling of a key greenhouse gas.

A second category of six taxa peaked in abundance during intermediate decay stages (DC3 and DC4; Figure 3 and Supplementary Figure S5). The abundance of five of these six taxa displayed an inexplicable, yet strong, positive relationship with C-content (%C; Figure 4). Yet only three of these taxa were correlated with %N and only a single taxon was associated with the C/N ratio. These correlations to N amongst the taxa displaying an intermediate pattern of abundance are in stark contrast to the strong relationships to %N and C/N ratio displayed for those taxa showing an early pattern of abundance. The weaker relationship between abundance and N continues through the late stage of abundance and into those taxa found only in the soil, suggesting that N has less of a role in dictating taxa abundance following the early stages of decomposition. Nine taxa increased in abundance during late stages of decomposition (DC3, DC4, DC5; Figure 3 and Supplementary Figure S6), and retained a strong presence in the soils. Four of these taxa had a weak positive relationship to N-content (%N), while only two displayed a weak, negative relationship with the C/N ratio (Figure 4).

Finally, a fourth pattern of abundance included five taxa that were common in soil, but were largely to entirely absent in CWD (Figure 3 and Supplementary Figure S7). These taxa likely have no role in CWD decomposition and show an inability to colonize CWD at any stage of decomposition. This contrasts to those taxa that display a late stage pattern of abundance that were common in DC 4 and 5 logs as well as the soil, suggesting that at least some taxa view the late stages of decomposition and soil as a continuum of habitat. Our study marks the first known to the authors to follow bacterial succession from CWD through to the soil stage (Baldrian and Banin, 2016), which represents a critical end phase of bacterial succession.

Though the abundance of taxa changed as decay progressed, our analysis of community structure in SD and CWD (excluding soils) revealed two broad primary assemblages: taxa that were most abundant during early stage of decomposition (SD, DC1, and DC2) and those more prominent during late stage of decomposition (DC3, DC4, and DC5). The bacterial community of the late stage became more closely representative of the soil microbiome, though an increased relative abundance of an uncharacterized group of Acidobacteria (DA052) and the presence of members of the phylum Solibacteres (Figure 1), along with all taxa showing a late pattern of abundance (Figure 3 and Supplementary Figure S7), highlight the uniqueness of the soil environment.

Of the environmental variables measured in this study (Supplementary Figure S8), nitrogen appears to be the main driver of bacterial succession, though this is most strongly evident in the early stages of decomposition. Wood in early stages of decay had a high C/N ratio, suggesting decomposition is N-limited (Osler and Sommerkorn, 2007). As a result, the high C/N ratio in the early stage of decomposition likely selects for nitrogen-fixing bacteria (Cowling and Merrill, 1966; Hoppe et al., 2014). However, as wood decomposition progressed, there was an increase in percent N, likely resulting from the combined effects of N-fixation, mineralization, and translocation. This shift in N abundance appears to demarcate the early stages of decomposition from the intermediate stages (Supplementary Figures S8c,d, and Figure 4), and as has been shown in other studies, may be responsible for bacterial community turnover (Cowling and Merrill, 1966; Wei and Kimmins, 1998; Brunner and Kimmins, 2003; Hoppe et al., 2014). Many bacterial groups increased in abundance along the chronosequence of wood decay (Figure 3 and Supplementary Figure S6), and *Pedosphaerales, Legionellales, Alicyclobacillaceae, Elusimicrobia* FAC88, *Sporichthya, Candidatus Solibacter*, and *Solibacterales* showed upsurges in population that correlated with increases in %N. This community might represent the late stage of succession where nitrogen is more readily available.

In contrast, the role of pH as a driver of succession is less clear in our study system. Across samples from all decay classes and soil, pH varied 100-fold from roughly 4.0 to 6.0 (Supplementary Figure S8f), but high variability across samples from within each decay class and the lack of significant differences between decay classes is not suggestive of any meaningful trend. This contrasts with findings from other studies where changes in pH have been found to drive changes in community structure (de Boer et al., 2010; Kielak et al., 2016b), but is consistent with other recent work in deadwood (Hoppe et al., 2014). *Acidobacteria* is one group of bacteria that have been found in dead wood and soils with low pH, and similar to Kielak et al. (2016a) we detected representatives of the *Acidobacteria* group in high abundance (Figure 1). We found that *Acidobacteria* were positively correlated with nitrogen fixers (*Rhizobiales* and *Burkholderiales*) and with decomposers (*Elusimicrobia* and *Xanthomonadales*). While the role of *Acidobacteria* in wood decomposition is not known, it is possible that this group contributes to wood decomposition through their expanded glycosyl hydrolase gene families (Kielak et al., 2016a), whose enzyme products play a role in the degradation of biomass such as cellulose and hemicellulose. While moisture content varied significantly over the chronosequence (Supplementary Figure S8a), moisture appears to be only a minor driver of bacterial succession correlating with taxon abundance in the early and intermediate stages of decomposition, but surprisingly having little influence on abundance in the late stages of decomposition or the soil (Figure 4).

While the presence and abundance of taxa in the early stages of decomposition seem to be largely dictated by the C/N ratio, and perhaps to a lesser extent pH and moisture, the later stages of decomposition showed a marked reduction in interaction with the environmental variables that we measured (Figure 4). Using moisture C/N, δN15, and pH, each taxon across the early, intermediate, and late patterns of abundance shown in Figure 4 interacted with 3.0, 1.0, and 0.75 environmental variables on average, respectively. This suggests that in the later stages of decomposition that abiotic variables become less deterministic in regards to community composition and succession. Our latent variable model suggests that positive interspecies interactions are more important in these later stages, such that community composition switches from being driven by abiotic factors to being driven more by biotic interactions.

Given our two distinct stages of decomposition, we investigated the community interspecific interactions via co-occurrence, aiming to identify the facilitation (beneficial interaction) and competition (negative interaction) among those microbes. One of the major challenges in investigating interspecific interactions consists in disentangling co-occurrence from abiotic correlation – when two apparently correlated species are, in fact, sharing a common dependence of a third environmental variable. We circumvent this problem by adopting a latent variable model that removes the effect of our environmental factors (wood characteristics) by evaluating the residual correlation. According to Warton et al. (2015) this approach provides a suitable way to identify biotic interactions such as competition and facilitation. However, the method is limited to the variables collected and unmeasured predictors may also impact this abiotic correlation.

Our results indicate that a more complex and interconnected community arises during the late successional stage (Figure 5, 6). Based on our model, results showed an increase in both the proportion (+9.3%) and the intensity (+62.3%) of positive interactions among orders of bacteria along the gradient of wood decay, independent of wood characteristics. As discussed above, it is during the late successional stage that bacteria showed a marked reduction in their interaction with the environmental variables that we measured (Figure 4). Thus, we appear to be detecting a shift whereby succession in the early stages is more broadly dictated by changes in wood chemistry and moisture and in the later stages community succession is more broadly dictated by species interactions. We hypothesize that this could result from the fact that the decomposition by some microorganisms leads to residual products of their metabolism that will be used as energy and nutrient sources by other microorganisms, establishing syntrophic relations between microbes, which maintain the balance of the biological community as a whole in the late stage of decomposition. Many studies on mixed cultures have reported that a combination of a cellulolytic bacteria with a non-cellulolytic bacteria result in a superior capacity of degradation and oxidation of cellulose (Hofsten et al., 1971; Dumova and Kruglov, 2009). Usually, degradation of cellulose releases cellobiose and glucose which feed a secondary group of microbes. Interestingly, this secondary group also helps the cellulose decomposers because these free sugars normally inhibit the degradation of cellulose (Ljungdahl and Eriksson, 1985).

Dini-Andreote et al. (2015) postulated that, within successional stages, community composition is initially governed by stochastic processes which become increasingly more deterministic as succession progresses. Here, we present evidence in support of Dini-Andreote et al. (2015), whereby the microbial community during the late stages of decomposition presented more positive interspecies interactions, which suggests an increase in the direct interactions between microbial populations. Moreover, we also found evidence that changes in wood characteristics favored some bacterial groups. These changes in the bacterial community are likely a consequence of the new ecological niches generated while decomposition advances.

The results of this research indicate that bacterial community succession is controlled by a complex and dynamic network of interactions shaped by a combination of abiotic wood variables and biotic interactions amongst the bacterial taxa. In order to understand more fully the role that bacteria are playing in decomposition, it will be critical to move beyond an understanding of taxa co-occurrence to a more complete understanding of the mechanisms underlying these interactions, which will require knowledge on bacterial metabolism. While we have some familiarity of the metabolism of some groups, such as the N-fixers, a more comprehensive metabolic picture will likely yield insight into functional redundancy, aiding comparisons across ecological systems. As prior research has shown that bacterial communities vary by tree species, soil types and ecosystems, it is imperative that we study CWD decomposition in numerous forest types and systems, before we know how generally applicable our results are. Finally, and likely most importantly, the process of microbial decomposition is controlled by an interaction between bacteria and fungi, and it is through a complete understanding of the patterns dictating this interaction that we will understand how decomposition of CWD occurs.

## Author Contributions

JS, BC, CG, and LF designed the research. BC and LF collected the field data. JS collected the molecular data. EK, ML, AS, RF, CG, and JS analyzed the data. EK, ML, AS, RF, and JS wrote the manuscript.

## Conflict of Interest Statement

The authors declare that the research was conducted in the absence of any commercial or financial relationships that could be construed as a potential conflict of interest.

## Footnotes

1 https://www.ebi.ac.uk/ena/
