# Supplementary material for: Wood Decay Characteristics and Interspecific Interactions Control Bacterial Community Succession in Populus grandidentata (Bigtooth Aspen)
Source: Front Microbiol. 2019 May 9;10:979. doi: 10.3389/fmicb.2019.00979 (PMC6520631; doi:10.3389/fmicb.2019.00979)
Supplement: Supplementary file 1 [file Data_Sheet_1.docx]

**Wood decay characteristics and interspecific interactions control bacterial community succession in *Populus grandidentata* (bigtooth aspen)**

Eiko E. Kuramae, Marcio F.A. Leite, Afnan K.A. Suleiman, Christopher M. Gough, Buck T. Castillo, Lewis Faller, Rima B. Franklin, John Syring

**Supplementary Material**

**Supplementary Table 1**. Primers used in DNA amplification and sequencing.

| \| **PRIMER** \| **FORWARD/ REVERSE** \| **SEQUENCE (5’ to 3’)** \| \| --- \| --- \| --- \| \| 799F \| Forward \| AACMGGATTAGATACCCKG \| \| 1193R \| Reverse \| GACGGGCGGTGWGTRCA \| |
| --- | --- | --- | --- | --- | --- | --- | --- | --- | --- |

**Supplementary Table 2**. Frequency table for the choice of the K number of clusters. For each criterion, the modal combinations of K are presented in bold. No criteria ever selected d = 0, i.e., a null model with no latent variables, and so that combination was removed from the table below. The best model was K = 2.

| K clusters | DICc | EAICc | EBICc | AICmed | BICmed |
| --- | --- | --- | --- | --- | --- |
| 1 | 9180.99 | - | - | 12069.72 | 13883.90 |
| 2 | 9003.23 | **10527.28** | **12345.33** | **2955.68** | **4773.74** |
| 3 | **8996.48** | 10533.8 | 12355.75 | 2969.72 | 4791.67 |
| 4 | 9032.20 | 10538.24 | 12364.08 | 3002.00 | 4827.83 |

**Supplementary Table 3**. Total number of sequences and Good’s sequence coverage (%).

| Sample | Number of sequences | Sequence Coverage (%) | Abbreviation | Treatment |
| --- | --- | --- | --- | --- |
| JVS.74 | 63,202 | 99.77 | SD | Standing Dead |
| JVS.63 | 59,913 | 99.75 | SD | Standing Dead |
| JVS.62 | 80,257 | 99.83 | SD | Standing Dead |
| JVS.28 | 30,807 | 99.55 | SD | Standing Dead |
| JVS.77 | 56,206 | 99.68 | DC1 | Decay Class 1 |
| JVS.64 | 65,020 | 99.74 | DC1 | Decay Class 1 |
| JVS.32 | 34,575 | 99.49 | DC1 | Decay Class 1 |
| JVS.30 | 38,917 | 99.54 | DC1 | Decay Class 1 |
| JVS.65 | 62,018 | 99.73 | DC2 | Decay Class 2 |
| JVS.58 | 29,762 | 99.37 | DC2 | Decay Class 2 |
| JVS.54 | 37,908 | 99.55 | DC2 | Decay Class 2 |
| JVS.33 | 38,945 | 99.56 | DC2 | Decay Class 2 |
| JVS.35 | 24,804 | 99.38 | DC3 | Decay Class 3 |
| JVS.55 | 37,097 | 99.39 | DC3 | Decay Class 3 |
| JVS.37 | 34,105 | 99.55 | DC3 | Decay Class 3 |
| JVS.36 | 44,143 | 99.58 | DC3 | Decay Class 3 |
| JVS.68 | 49,664 | 99.54 | DC4 | Decay Class 4 |
| JVS.42 | 51,468 | 99.58 | DC4 | Decay Class 4 |
| JVS.41 | 39,372 | 99.39 | DC4 | Decay Class 4 |
| JVS.40 | 29,150 | 99.37 | DC4 | Decay Class 4 |
| JVS.72 | 48,015 | 99.57 | DC5 | Decay Class 5 |
| JVS.69 | 47,882 | 99.53 | DC5 | Decay Class 5 |
| JVS.45 | 42,158 | 99.53 | DC5 | Decay Class 5 |
| JVS.44 | 37,774 | 99.37 | DC5 | Decay Class 5 |
| JVS.61 | 59,847 | 99.63 | S | Soil |
| JVS.49 | 46727 | 99.64 | S | Soil |
| JVS.48 | 46729 | 99.61 | S | Soil |


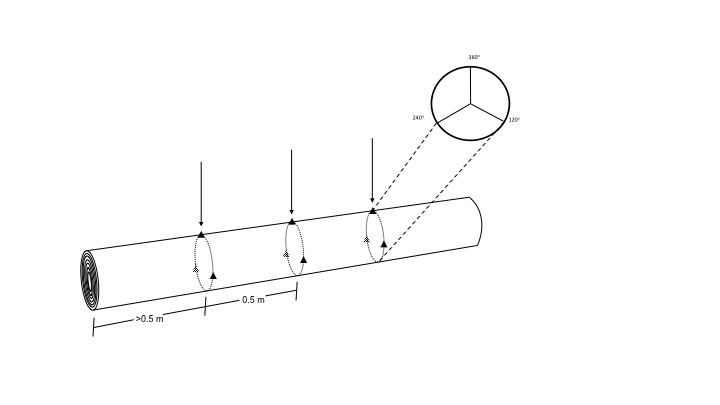


**Supplementary Figure S1.** CWD sampling design illustrating the position of drilling points along the length of each downed log. Arrows and dotted lines indicate the location of each of three sampling positions. At each sampling position, three cores were drilled (triangles) at 120°, 240° and 360° giving a total of 9 cores per log. See text for details.


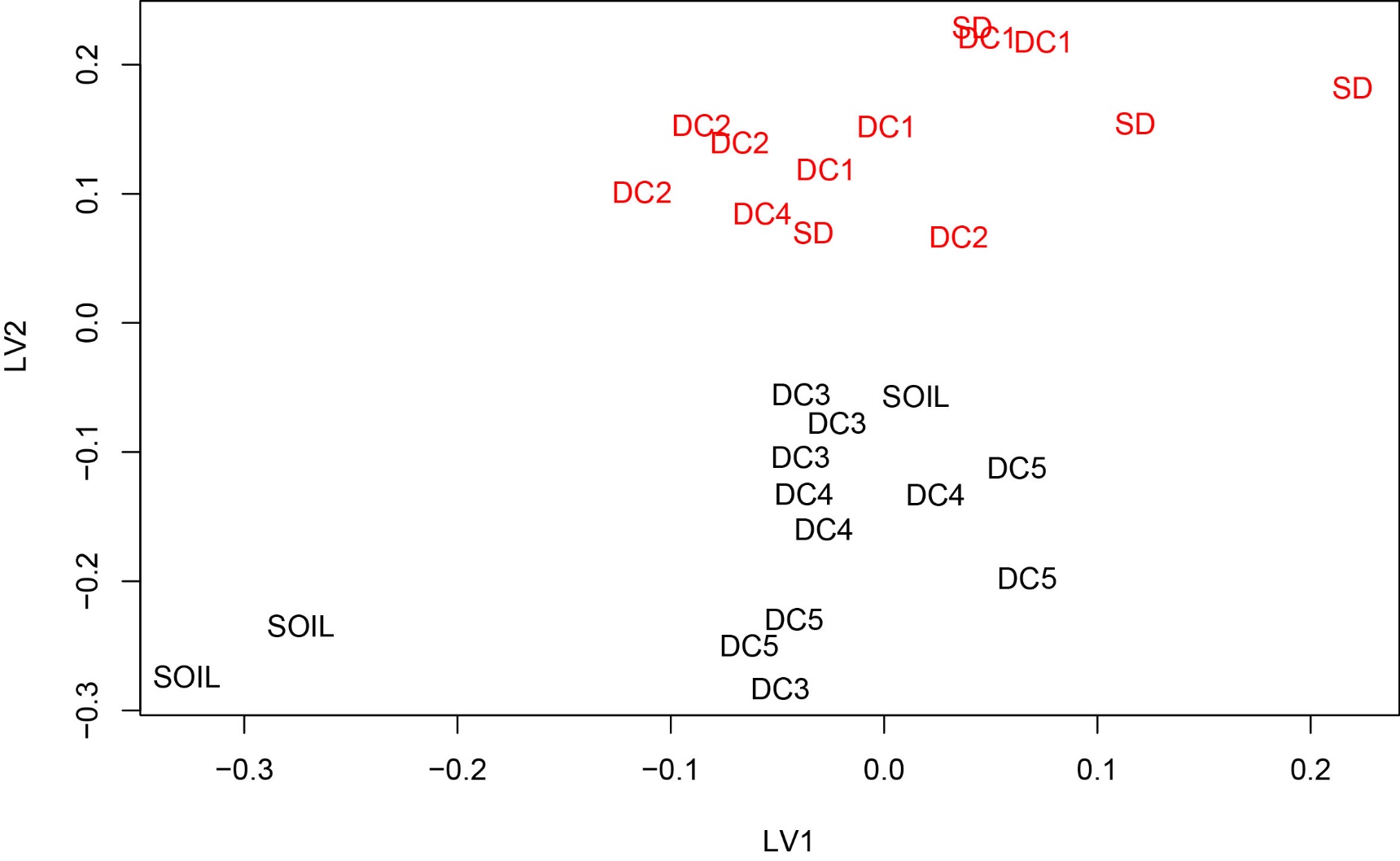


**Supplementary Figure S2.**  Ordination plots for the dataset using CORAL with 2 latent variables, K = 2 clusters, and site effects. The clusters are represented by colors and the abbreviations represent CWD sample types including standing dead (SD) and decay classes 1 through 5 (DC1 to DC 5.

*
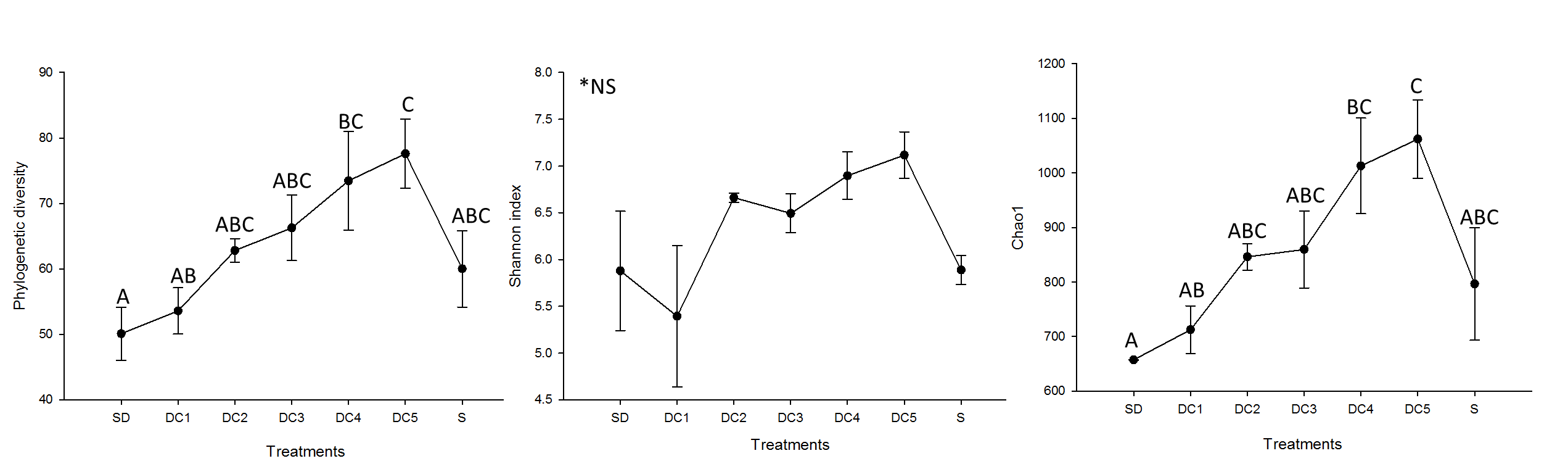
*

**Supplementary Figure S3**. Phylogenetic Diversity, Shannon Index, and Chao 1 estimator of bacterial community diversity across all stages of decay in CWD (SD, DC1, DC2, DC3, DC4, DC5) and soil (S). Means marked by the same letter did not differ statistically (ANOVA followed by Tukey’s *post hoc* test with α=0.05). NS: not significant.


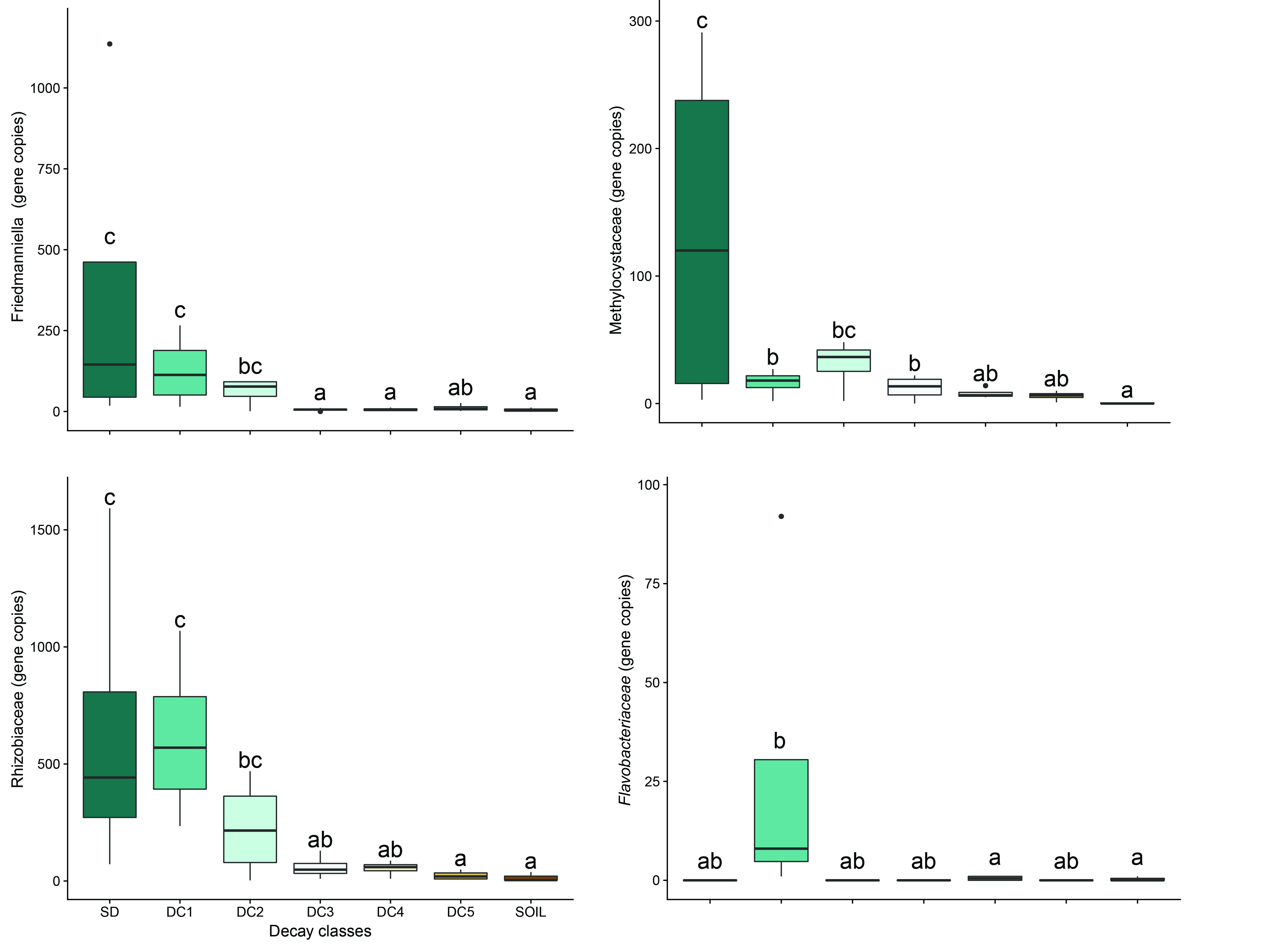


**Supplementary Figure S4**. Bacterial groups with peak abundances in the early stages of decomposition. These bacterial taxa were weakly (p<0.06) affected by the decay status of their host logs or soil conditions. Boxes represent the 25 to 75 percentiles and display the median values (bold line). The whiskers highlight the maximum and minimum values (non-outlier range). Decay classes followed by the same letter do not differ from each other according to the Tukey-Kramer test with α=0.05.


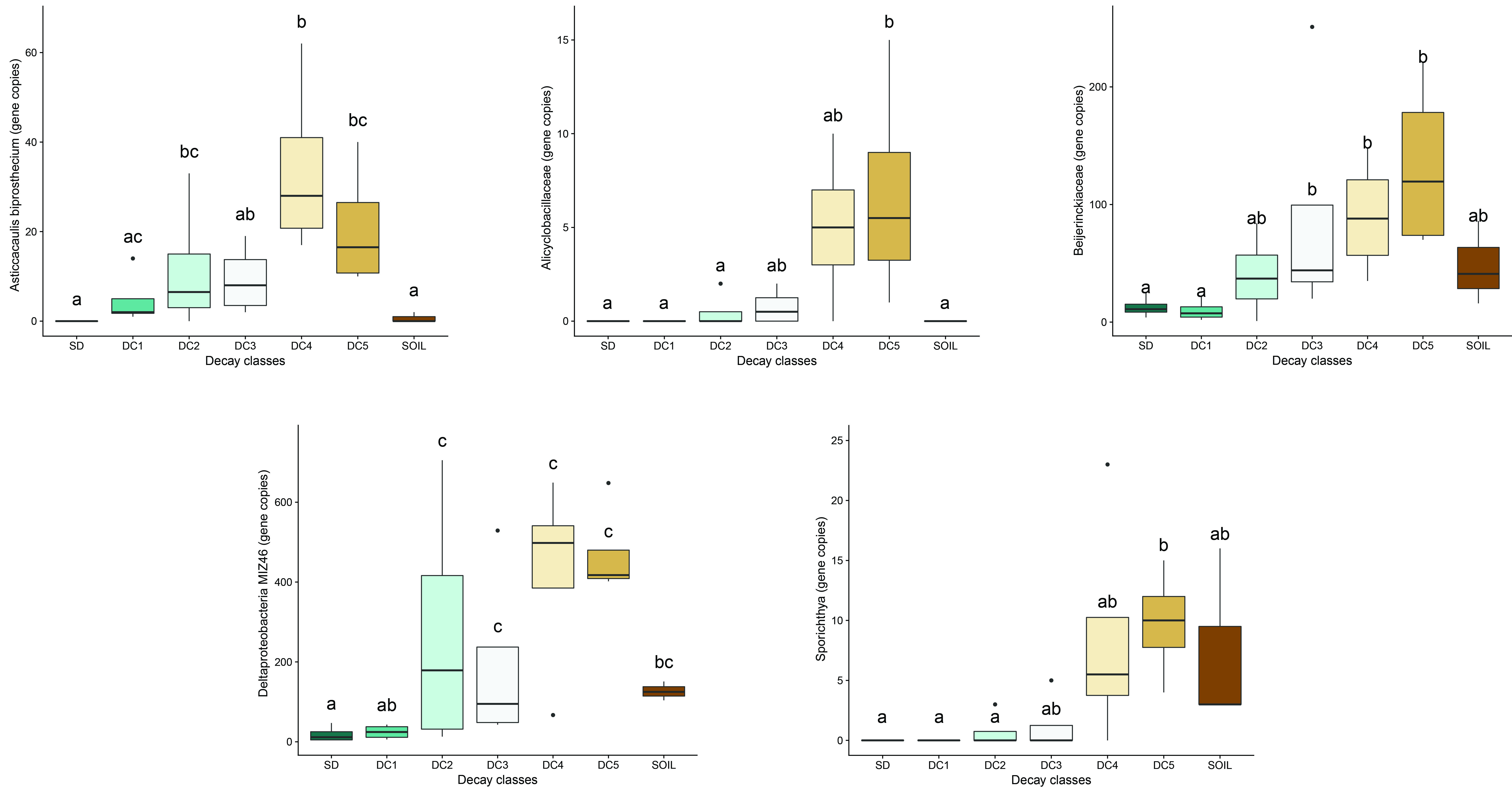


**Supplementary Figure S5**. Bacterial groups whose abundance peaked in the mid stages of wood decay. These bacterial taxa were weakly (p<0.06) affected by the decay status of their host logs or soil conditions. Boxes represent the 25 to 75 percentiles and display the median values (bold line). The whiskers highlight the maximum and minimum values (non-outlier range). Decay classes followed by the same letter do not differ from each other according to the Tukey-Kramer test with α=0.05.


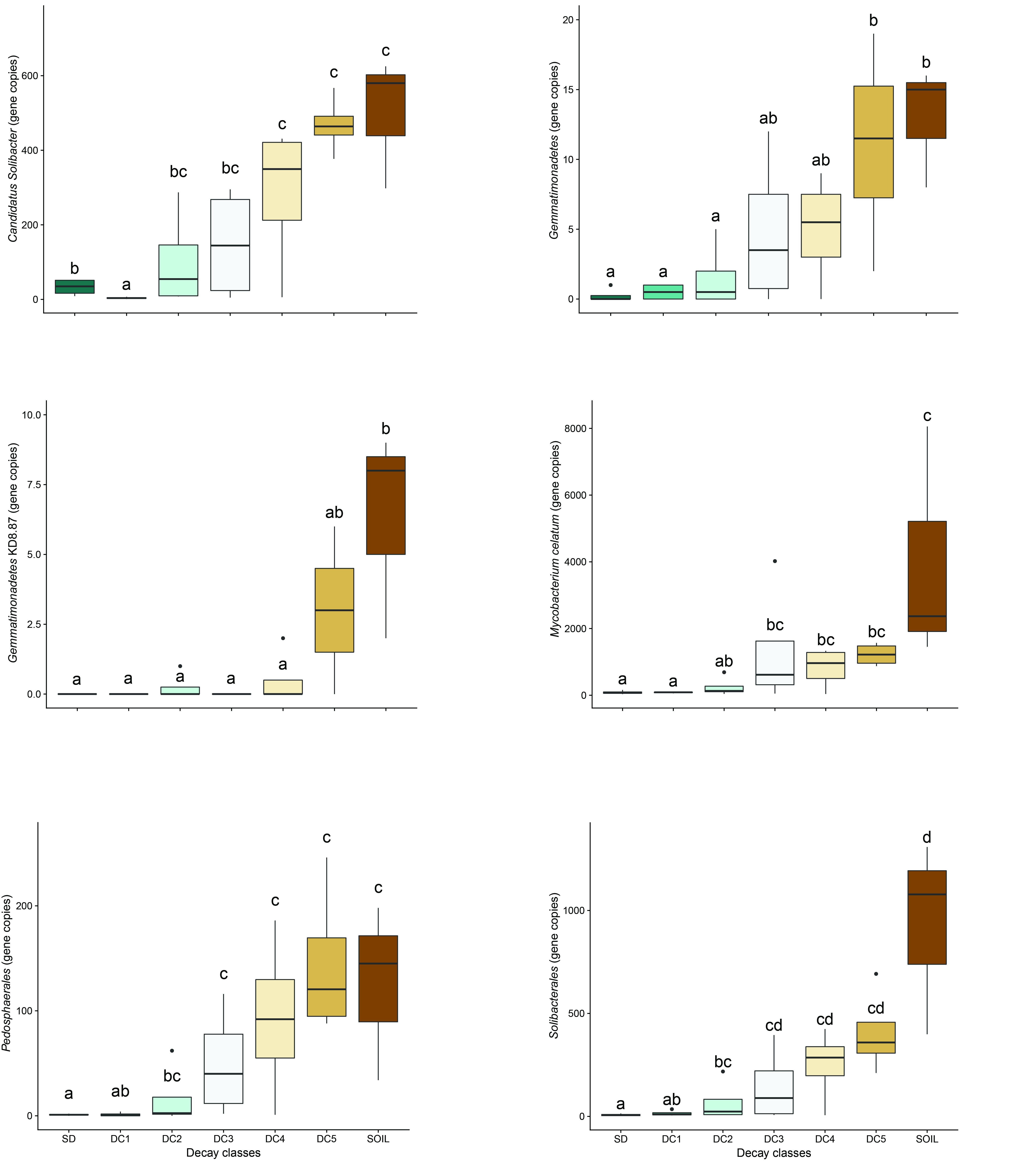


**Supplementary Figure S6**. Bacterial groups with peak abundances in the late stages of decomposition. These bacterial taxa were weakly (p<0.06) affected by the decay status of their host logs or soil conditions. Boxes represent the 25 to 75 percentiles and display the median values (bold line). The whiskers highlight the maximum and minimum values (non-outlier range). Decay classes followed by the same letter do not differ from each other according to the Tukey-Kramer test with α=0.05.


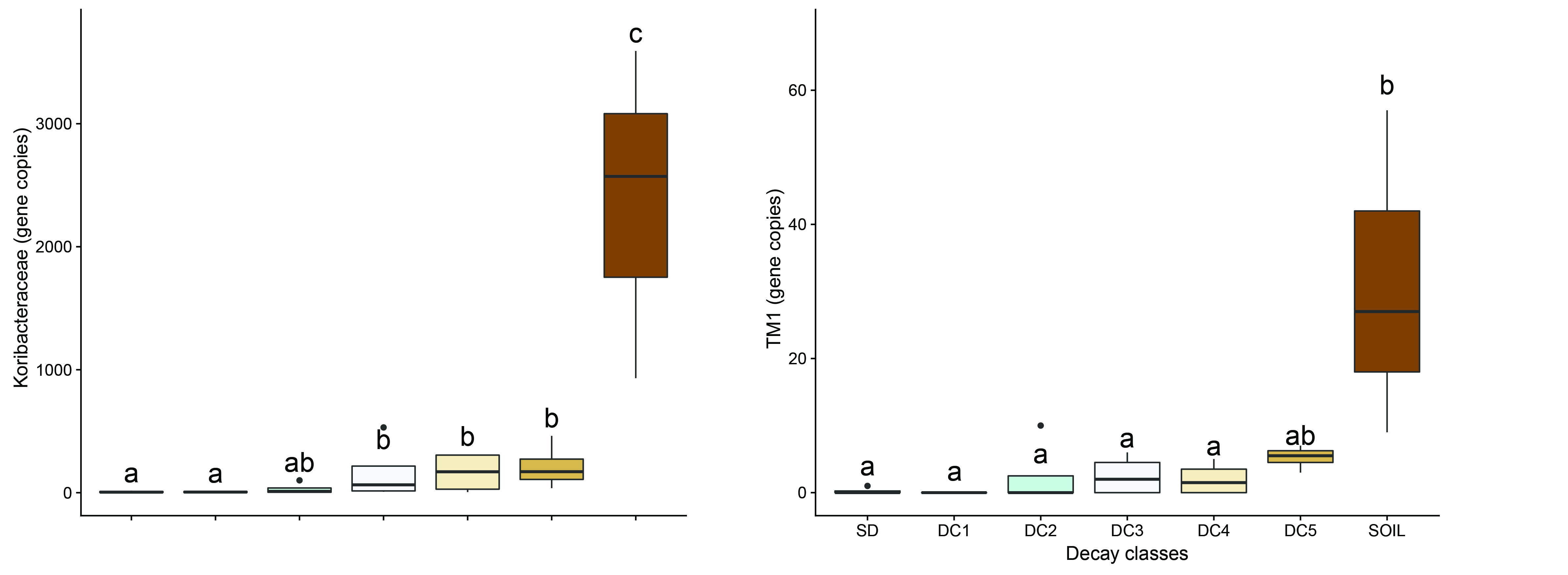


**Supplementary Figure S7**. Bacterial groups which are abundant in soil but are rare in all stages of wood decay. These bacterial taxa were weakly (p<0.06) affected by the decay status of their host logs or soil conditions. Boxes represent the 25 to 75 percentiles and display the median values (bold line). The whiskers highlight the maximum and minimum values (non-outlier range). Decay classes followed by the same letter do not differ from each other according to the Tukey-Kramer test with α=0.05.

**
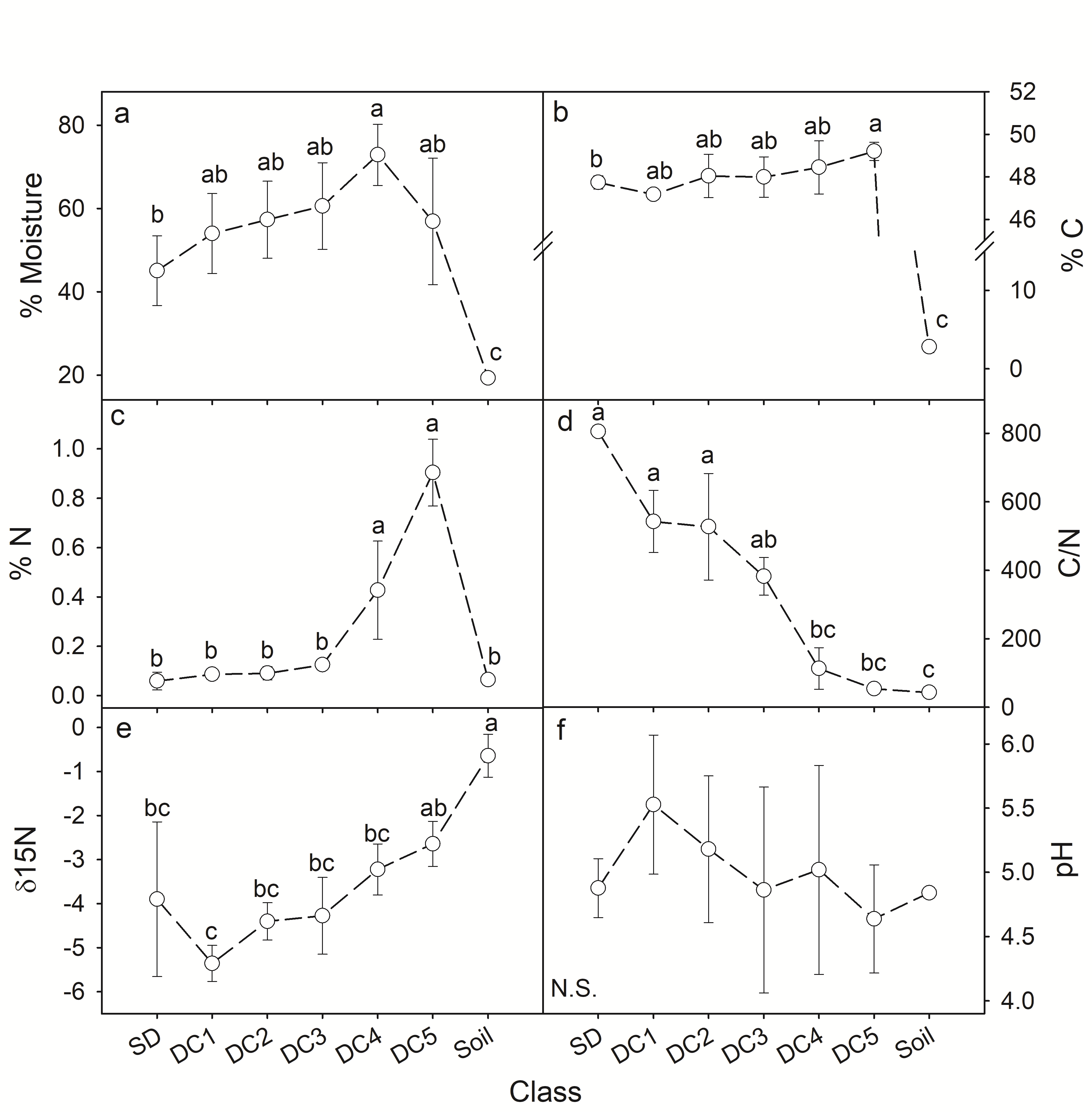
**

**Supplementary Figure S8.** Changes in the water availability and chemical composition of standing dead (SD), through wood decay classes (DC1= Decay Class 1, DC2= Decay Class 2, DC3= Decay Class 3, DC4= Decay Class 4, DC5= Decay Class 5), and soil. Means for each class are depicted as open circles and whiskers represent standard deviations. Means marked by the same letter did not differ statistically (ANOVA followed by Tukey’s *post hoc* test with α=0.05). NS: not significant.


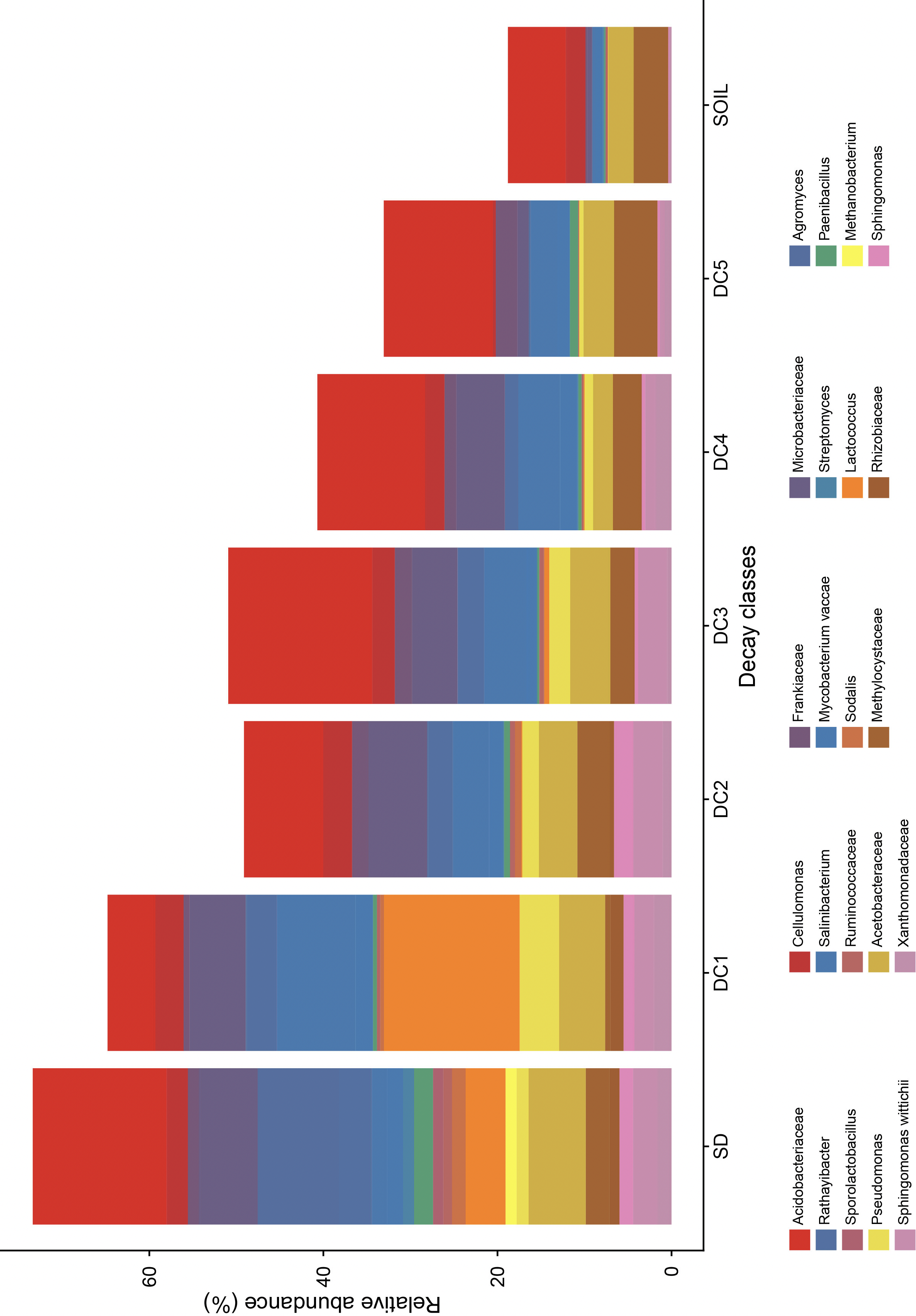


**Supplementary Figure S9**. Percentage of shared taxa between standing dead trees and remaining decay classes – soil. Abundances are relative to the total bacterial pool within each class. Taxa not shared with standing dead trees are excluded as are rare taxa which constitute less than 5% relative abundance.


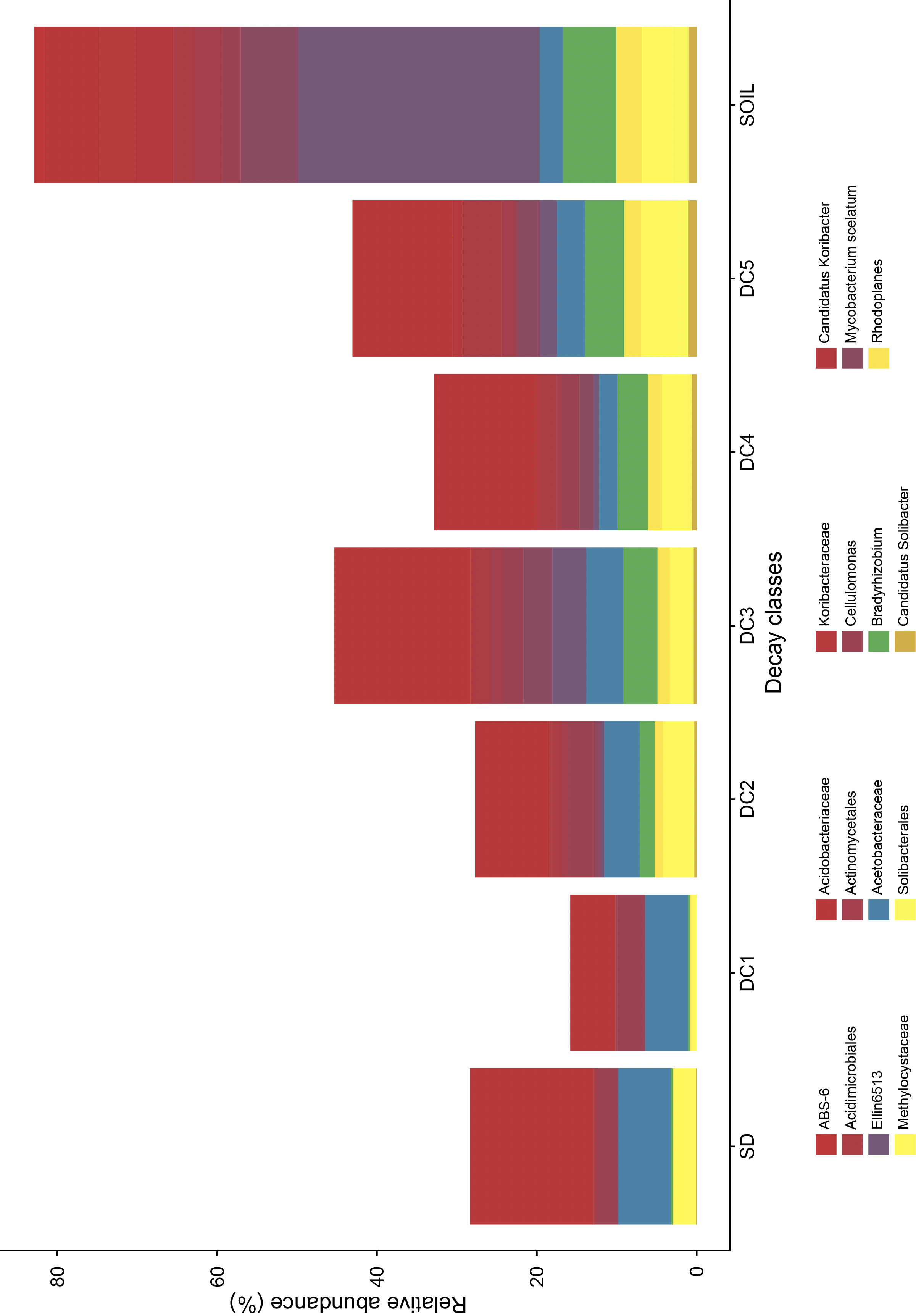


**Supplementary Figure S10**. Percentage of shared taxa between soil and remaining decay classes. Abundances are relative to the total bacterial pool within each class. Taxa not shared with soil are excluded as are rare taxa which constitute less than 5% relative abundance.
